# A Novel Mouse c-*fos* Intronic Promoter That Responds to CREB and AP-1 Is Developmentally Regulated *In Vivo*

**DOI:** 10.1371/journal.pone.0011235

**Published:** 2010-06-21

**Authors:** Vincent Coulon, Karim Chebli, Patricia Cavelier, Jean-Marie Blanchard

**Affiliations:** Institut de Génétique Moléculaire de Montpellier, Centre National de la Recherche Scientifique, Université Montpellier 2, Université Montpellier 1, Montpellier, France; The University of Hong Kong, China

## Abstract

**Background:**

The c-*fos* proto-oncogene is an archetype for rapid and integrative transcriptional activation. Innumerable studies have focused on the canonical promoter, located upstream from the transcriptional start site. However, several regulatory sequences have been found in the first intron.

**Methodology/Principal Findings:**

Here we describe an extremely conserved region in c-*fos* first intron that contains a putative TATA box, and functional TRE and CRE sites. This fragment drives reporter gene activation in fibroblasts, which is enhanced by increasing intracellular calcium and cAMP and by cotransfection of CREB or c-Fos/c-Jun expression vectors. We produced transgenic mice expressing a lacZ reporter controlled by the intronic promoter. Lac Z expression of this promoter is restricted to the developing central nervous system (CNS) and the mesenchyme of developing mammary buds in embryos 12.5 days post-conception, and to brain tissue in adults. RT-QPCR analysis of tissue mRNA, including the anlage of the mammary gland and the CNS, confirms the existence of a novel, nested mRNA initiated in the first intron.

**Conclusions/Significance:**

Our results provide evidence for a novel, developmentally regulated promoter in the first intron of the c-*fos* gene.

## Introduction

The c-*fos* proto-oncogene product, c-Fos, dimerizes with members of the Jun family to form the transcription factor AP-1, which regulates a wide array of genes in response to many stimuli [1]. c-*fos* gene activation has been extensively studied because it exemplifies the rapid, transient response to extracellular stimuli. c-*fos* is kept silent in most cell types but is robustly induced by a wide range of agents [2] including: mitogens [3], cellular stresses such as UV irradiation [4] and mechanical stretch [5], synaptic stimulation [6], and lymphocyte activation [7]. Induction is usually transient: c-*fos* mRNA accumulation peaks 15–30 min post-induction and disappears after 1 h, reflecting both transcriptional shut-off and mRNA destabilization [8], [9].

These features make c-*fos* an exquisite model for studies on transcriptional control, and the regulatory sequences in its promoter have been extensively studied. These include sites required for the response to cytokines (SIE, [10]), serum growth factors (SRE, [8], [11], [12]), calcium and cAMP (CRE, [13], [14]). Transcription factors that bind these elements have been identified: STAT1 and 3 (SIE) [15], SRF and TCF (SRE) [16], [17], and members of the CREB/ATF family (CRE, reviewed in [18]).

However, c-*fos* expression *in vivo* cannot be explained by a one signal/one transcription factor/one promoter element reductionism. Indeed, Robertson and coworkers showed in transgenic mice that c-*fos* regulation could only be faithfully mimicked by a reporter controlled by the whole gene sequence [19]. Moreover, using mutants of the SIE, SRE, FAP and CRE sequences, they showed that inactivation of any of these sites led to a dramatic loss of basal and induced activity [19]. These data are consistent with results of Herrera and coworkers, showing that a nucleosome settles in the middle of the promoter and persists throughout the gene activation cycle [20]. Taken together, this suggests that higher order complexes involving specific transcription activators, coactivators and the so-called « basal » transcriptional apparatus integrate diverse signals to elaborate a controlled response.

Moreover, studies from our laboratory and others identified intragenic transcription control regions. First, the 5′ part of the first intron contains sequences required for a transcription elongation block that occurs 385 bp downstream the start site *in vitro* [21] and in cells [22]. This blockade is relieved by calcium signalling [23]–[25] through a novel pathway [22], and contributes to rapid activation in this context. Second, a Fos Intragenic Regulatory Element (FIRE) was identified [26] that appears to be independent of the elongation block [21], [22].

In addition, DNase I-hypersensitive sites in the c-*fos* gene map to the SRE and the transcription start site (TSS), and to two intragenic positions, at +200 and +700 relative to the TSS [27], that presumably correspond to regulatory sites. The +200 region corresponds to the FIRE sequence [26], while the +700 site maps to the conserved region described in this work.

c-*fos* expression has been followed during mouse development using *in situ* hybridization on frozen embryo sections. c-*fos* mRNA was first detected in developing bone and cartilage in E17–E18 embryos [28]. Accordingly, c-*fos* gene knockout mice exhibit a severe bone development defect, osteopetrosis [29], [30], due to a defect in osteoclast differentiation [31]. The lack of more widespread phenotypes in c-*fos* null mice indicates that, in spite of its apparently ubiquitous role in proliferation and differentiation of cultured cells, c-Fos functions can largely be compensated by other Fos family members.

Here we show that c-*fos* first intron contains a region that is highly conserved from Xenopus to man, and contains binding sites for TBP (TATA box), along with the AP-1 and CREB families of transcription factors. This region promotes luciferase reporter gene expression in fibroblasts. Moreover, this promoter activity is enhanced by activating cAMP and Ca^2+^ signaling pathways, as well as by ectopic expression of CREB, c-Fos and c-Jun. To test its activity *in vivo*, we produced transgenic mice carrying a construct in which the intronic sequence controls expression of LacZ. Transgenic embryos show LacZ expression in various areas of the CNS throughout development, and in the developing mammary gland at days 12.5 to 13.5 p.c. We confirmed by RT-QPCR that this novel promoter activity actually produces a messenger RNA *in vivo*. Our results suggest that previous data on c-*fos* regulation should be re-evaluated in light of the existence of this new promoter.

## Results

### Sequences within the 3′ part of c-*fos* first intron have been conserved through evolution

**Figure 1:**
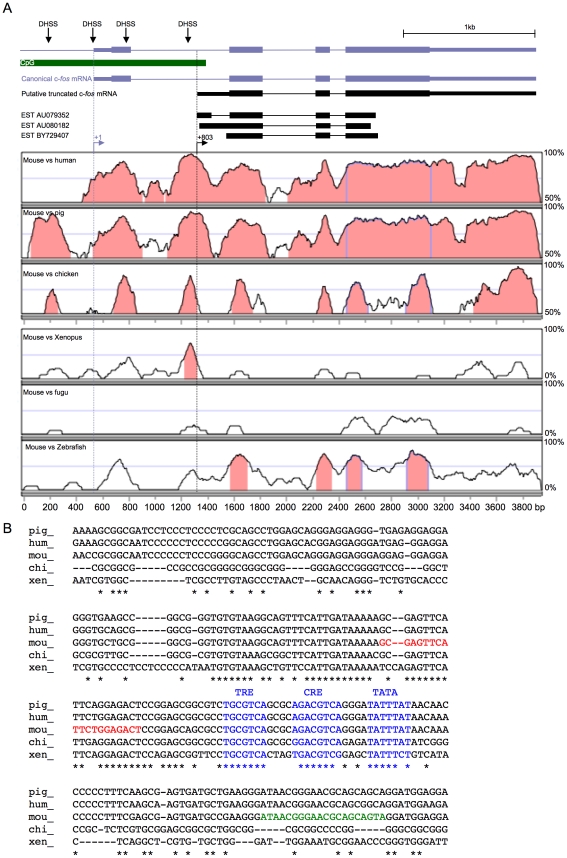
An extremely conserved region in c-*fos* first intron: evidence for a nested promoter. A. c-*fos* gene conservation profile between the mouse gene and 6 other indicated species (VISTA genome server). The baseline corresponds to 50% identity for human, pig and chicken genomes, and to 0% identity for xenopus, fugu, and zebrafish genomes in 100 bp windows. Peaks culminating at more than 70% identity (in 100 bp windows) are painted in pink by the software. Note that the 3′ half of first intron is more conserved than surrounding exons in the four top species. Confirmed start site for canonical mRNA and putative intronic mRNA appear respectively as blue and black broken arrows. A CpG island (green box) extends from the canonical promoter to the putative intronic promoter. Blue boxes represent exons, black mRNAs correspond to the putative mRNA starting in the conserved region and three ESTs previously sequenced and mapping to the same region (UCSC genome server). DNase I hypersensitive sites from Renz et al. are shown as black downward arrows. B. Nucleotide alignment of the most conserved part of c-*fos* first intron (from +619 to +849 relative to mouse canonical TSS) from 5 species (pig, human, mouse, chicken, xenopus). Asterisks depict nucleotides identical in all 5 species. Conservation culminates in motifs resembling a TRE, a CRE, and a TATA box (shown in blue). Red and green sequences respectively correspond to primers 1 and 2 used in figure 6.

We compared c-*fos* mouse genomic sequences with those from *Homo sapiens*, *Sus scrofa*, *Gallus gallus*, *Xenopus laevis* [32], *Fugu rubripes* and *Danio rerio* (see *Methods* section for accession numbers), using the VISTA genome server. We noticed an extremely conserved region in intron 1, even more conserved than c-*fos* exons (fig. 1A). A nucleotide alignment of the 3′ part of this c-*fos* intron 1 shows that between nucleotides +619 to +849 relative to the murine TSS, 43% nucleotides are strictly identical in five species (fig. 1B). Such a degree of conservation suggests high selective pressure. In addition, this intron spans ca. 400 bp in the c-*fos* ortholog from *Fugu rubripes*, an organism which has a very compact genome with rare introns. Strikingly, the sequences from *Fugu rubripes* and *Mus musculus* are 50% identical (when aligning nucleotides +692 to +792 relative to the mouse gene, not shown). Taken together, these observations suggest that this part of the c-*fos* gene has an important regulatory function. In addition, three ESTs (AU079352, AU080182 and BY729407) from mouse adult brain and embryo spinal cord map in this intronic region (fig. 1A), suggesting that it contains an alternative promoter with a preference for neural tissue.

Strikingly, this region harbors DNA sequences that resemble consensus binding sequences for AP-1 (TRE) and CREB (CRE) families of bZIP transcription factors, immediately upstream of a putative TATA box (fig. 1B, in blue). These sites show strong conservation from Xenopus to man and were detected at high stringency by the Alibaba program (see *Methods* section) that predicts transcription factor binding sites.

Consistent with this region being a putative promoter, sequence conservation is high upstream of the TATA box, and drops sharply just after it. In the Neural Network Promoter Prediction program (see *Methods* section), the region encompassing these conserved sites obtained a score of 0.92, where the score for the canonical promoter, defined as nucleotides −360 to +1, was 1.0. These observations strongly suggest that this intronic region could be a functional promoter.

### Intronic CRE and TRE sites bind transcription factors of the CREB and AP-1 families *in vitro*

**Figure 2:**
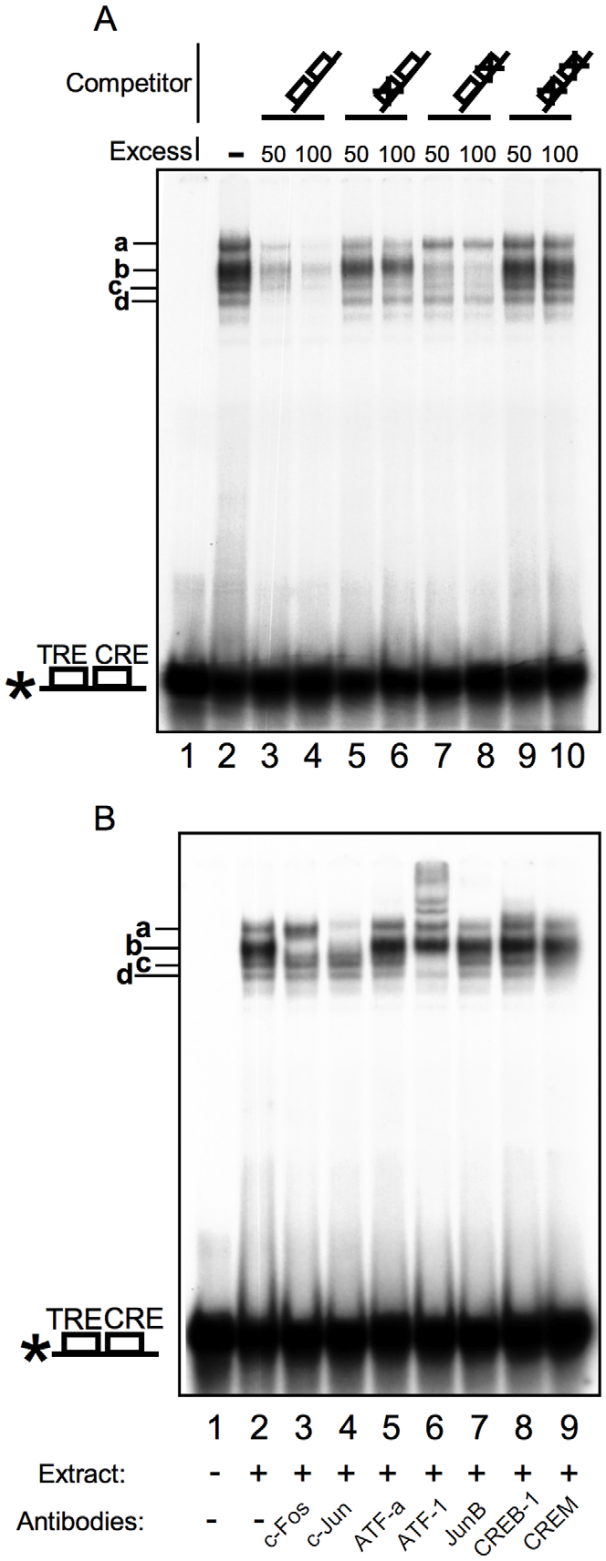
The intronic TRE and CRE sites bind members of the CREB and AP-1 families of transcription factors. EMSA using HeLa nuclear extracts and probes corresponding to the TRE and CRE sites of c-*fos* first intron. A. Competition analysis with non-radioactive probes wt (lanes 3, 4) or mutated on the TRE (lanes 5, 6), the CRE (lanes 7, 8), or both (lanes 9, 10). Lane 1 shows the probe without extract, lane 2 with extract but without competing cold probe. B. Disruption or supershift of the protein/DNA complexes. The probe (lane 1) was incubated with HeLa nuclear extract (lanes 2 to 9) and antibodies to c-Fos (lane 3), c-Jun (lane 4), ATF-a (lane 5), ATF-1 (lane 6), JunB (lane 7), CREB-1 (lane 8), and CREM (lane 9).

Given its high degree of conservation and similarity to TRE and CRE sites, we first tested its ability to recruit transcription factors *in vitro*. Multiple complexes were observed in an Electrophoretic Mobility Shift Assay using Hela cell nuclear extracts and a radioactive probe containing both putative sites (fig. 2A, lane 2). Competition experiments using an excess of unlabeled wt probe blocked complex formation (lanes 3, 4). Similarly, competing DNA mutated in a single site (lanes 5, 6, 7, 8) showed that band **b** corresponds to complexes on the TRE, while band **a** represents binding to the CRE. The bands **c** and **d** are not fully competed and may reflect binding activities capable of recognizing either site. Competition with an excess of the double mutant probe (lanes 9 and 10) had no effect, showing that they are specific for the TRE and CRE.

We then used antibodies specific for the AP-1 and CREB families of transcription factors to confirm that the complexes obtained in fig. 2A contained these factors. Antibodies to ATFa (fig. 2B, lane 5) and CREM (lane 9) had no detectable effect on the complexes. Antibodies to c-Fos (lane 3) and c-Jun (lane 4) disrupted the **b**, and **a** plus **b** complexes, respectively. Anti-JunB (lane 7) also reduced complex **a**. Antibodies to ATF-1 (lane 6) led to multiple supershifted complexes and the loss of complexes **c** and **d**. Anti-CREB-1 (lane 8) led to a weak supershift of complex **a**. No effect was seen in the absence of nuclear extract (not shown). Hence, consistent with the competition data, band **b** is likely to be AP-1 (c-Jun/c-Fos dimers) on the TRE site, while band **a** corresponds to CREB and c-Jun-containing complexes on the CRE site. Bands **c** and **d** seem to represent binding by ATF-1-containing complexes.

Thus the TRE and CRE sites are genuine *in vitro* binding sites for the AP-1 and CREB families of transcription factors.

### The highly conserved intronic region is sufficient to drive luciferase expression in transfected cells

**Figure 3:**
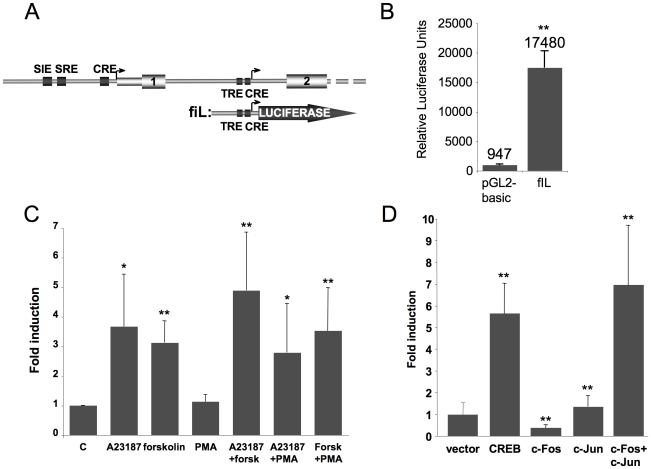
The c-*fos* conserved intronic region drives luciferase expression and responds to the CREB and AP-1 pathways. A. 5′ part of mouse c-*fos* gene (up) and our reporter construct (bottom), fiL (*fos* intron Luciferase). B. The *fos* intronic region suffices to drive Luciferase activity in NIH3T3 cells in transient transfection. The promoterless pGL2 basic vector background activity is shown as a control. Error bars represent standard deviation, n = 7. C. The fIL construct responds to the calcium and cAMP pathways, but not to PMA. NIH3T3 transfected with fiL were treated with the indicated drugs. Error bars correspond to standard deviation, n = 6. D. Co-transfection with CREB or AP-1 expression vectors stimulates fIL promoter activity. Error bars correspond to standard deviation, n = 5. Statistical analysis of variance (ANOVA) was performed (*: p<0.05; **: p<0.01 relative to control).

We designed the fiL reporter construct (*fos* intron Luciferase, fig. 3A, see *methods*) to check for intronic promoter activity in transient transfection assays. Interestingly, this construct gives significantly higher basal luciferase activity than the promoterless vector (pGL2-basic) in NIH 3T3 cells (fig. 3B), along with MEFs and CCL39 cells (Mouse Embryo Fibroblasts, Chinese Hamster Lung Fibroblasts; not shown). Furthermore, this activity is stimulated by agonists that elevate intracellular calcium and cAMP levels, the calcium ionophore A23187 and forskolin: they both lead to a 3-fold increase in promoter activity (fig. 3C) that was not observed with the promoter-less vector (pGL2-basic, not shown). The phorbol ester PMA did not enhance the intronic promoter activity, alone or together with A23187 and forskolin. Similarly, the combination of A23187 and forskolin did not increase intron promoter activity much over that seen with forskolin alone.

Since the region of interest contains CRE and TRE sites and responds to calcium and cAMP, we checked whether its activity was stimulated by expression vectors for AP-1 or CREB, which mediate responses to activation of calcium and cAMP pathways. While transfection of FosB, Fra-1, Fra-2, JunB or JunD did not stimulate the fiL reporter gene (not shown), the CREB expression vector stimulated fIL activity 6-fold (fig. 3D). Similarly, cotransfection of c-Fos and c-Jun expression vectors led to a 7-fold stimulation, not seen with either vector alone (fig. 3D).

Considering the *in vitro* effect of CREB and AP-1 factors on intron 1-driven transcription, we felt compelled to test the activity of the intronic promoter *in vivo* using transgenic mice.

### The c-*fos* intronic promoter directs β-galactosidase expression in specific stages and tissues of mice development

**Figure 4:**
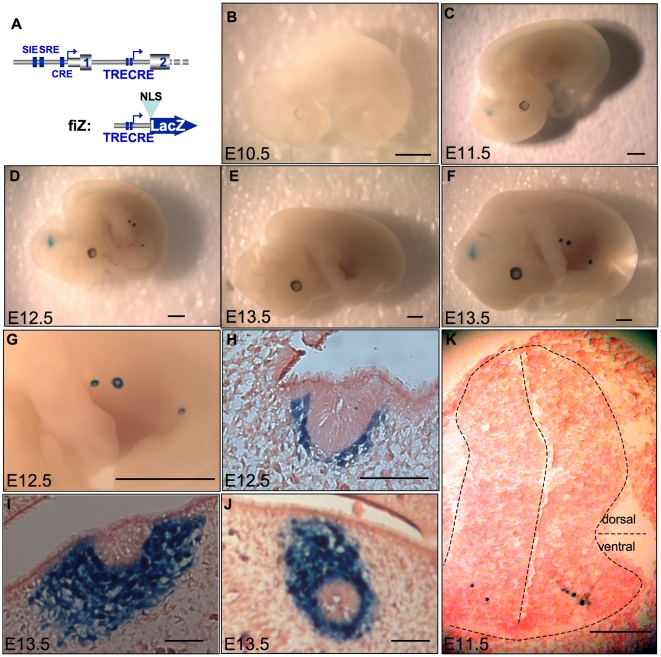
Transgenic analysis of the putative promoter shows expression restricted to the spinal cord and mammary bud of mouse embryos. A. 5′ part of mouse c-*fos* gene (up) and our NLS-containing, betagalactosidase reporter construct (bottom), fiZ (*fos* intron lacZ). 7 Transgenic mouse lines were created with fiZ and transgenic embryos were stained for betagalactosidase activity. Here are shown transgenic embryos from mouse line #60 at day 10.5 (B), 11.5 (C, K), 12.5 (D, G, H), and 13.5 (F, I, J). B, C, D, F: whole-mount embryos showing the spinal cord staining starting day 11.5 p. c. and the mammary gland anlage staining starting 12.5 p. c. G. Close-up view of the developing mammary buds showing the stained ring corresponding to mesenchymal cells. H, I, J: sagittal frozen sections showing the nuclear staining in the mesenchymal part of the mammary bud, but not in the central, epithelial part. K. Transverse frozen section on a 11.5 d. p. c. embryo showing the extremely restricted, ventral spinal cord staining. E: wild-type, e13.5 embryo as a control. Scale bars: 1 mm (B, C, D, E, F, G) then 150 µm (H, I, J, K).

In order to identify when and where the c-*fos* intronic promoter is active in mouse embryos, we constructed the fiZ transgene (*fos* intron LacZ, fig. 4A, see *methods*). As in the case of the fiL construct described before, this transgene contains no previously described promoter sequence. Fusing β-galactosidase to a nuclear localization signal (NLS) allows us to discriminate between it and the endogenous, cytoplasmic β-galactosidase activity [33].

The transgenesis procedure yielded 7 founders, 5 of which expressed LacZ activity during embryonic development. The expression territories were remarkably restricted. In addition to some ectopic expression due to random transgene insertion sites (Table 1), the fiZ embryos showed expression in the mesenchymal part of the mammary gland anlage from E12.5 to E13.5 (4/5 mouse lines, see fig. 4D, F, G–J). Another preferential site of expression was the developing CNS: 3/5 mouse lines showed transient fiZ expression in the spinal cord and hindbrain at E11.5 to E12.5 (fig. 4C–D). Embryonic sections revealed that β-galactosidase activity was nuclear (fig.4H–K), and thus resulting from expression of the fiZ transgene, not endogenous β-galactosidase. In addition, transverse sections showed that, in the spinal cord, only a few cells per section expressed the transgene (fig. 4K). These cells are located in the ventral part of the mantle layer, called the basal plate, that contains developing motor neurons. The linear aspect of the staining along the rostrocaudal axis of embryos also suggests that it represents a functional population of neurons from the same motor column.

**Table 1:** Frequency of occurrence of expression patterns for the fiZ transgene.

*Stage*	*Tissue*	*Frequency*
**Embryo**	Eye lens	2/5
	Vibrissae placodes	1/5
	Forebrain	1/5
	Forelimb ectoderm	1/5
	Forelimb AER	1/5
	**Mammary gland mesenchyme**	**4/5**
	**Spinal cord**	**3/5**
	**hindbrain**	**4/5**
**Newborn and adult**	**Brain** (different patterns)	**3/5**
	Spinal cord	0/5
	Other tissues	0/5

Expression territories which appear in more than two mouse lines are shown in boldface, the others being considered as possibly due to the influence of fiZ transgene insertion site. AER stands for Apical Ectodermal Ridge. Different expression sites in the newborn/adult brain are scored together as “brain”, but since they were not consistent in different transgenic lines, the precise regions have not been characterized in detail.

**Figure 5:**
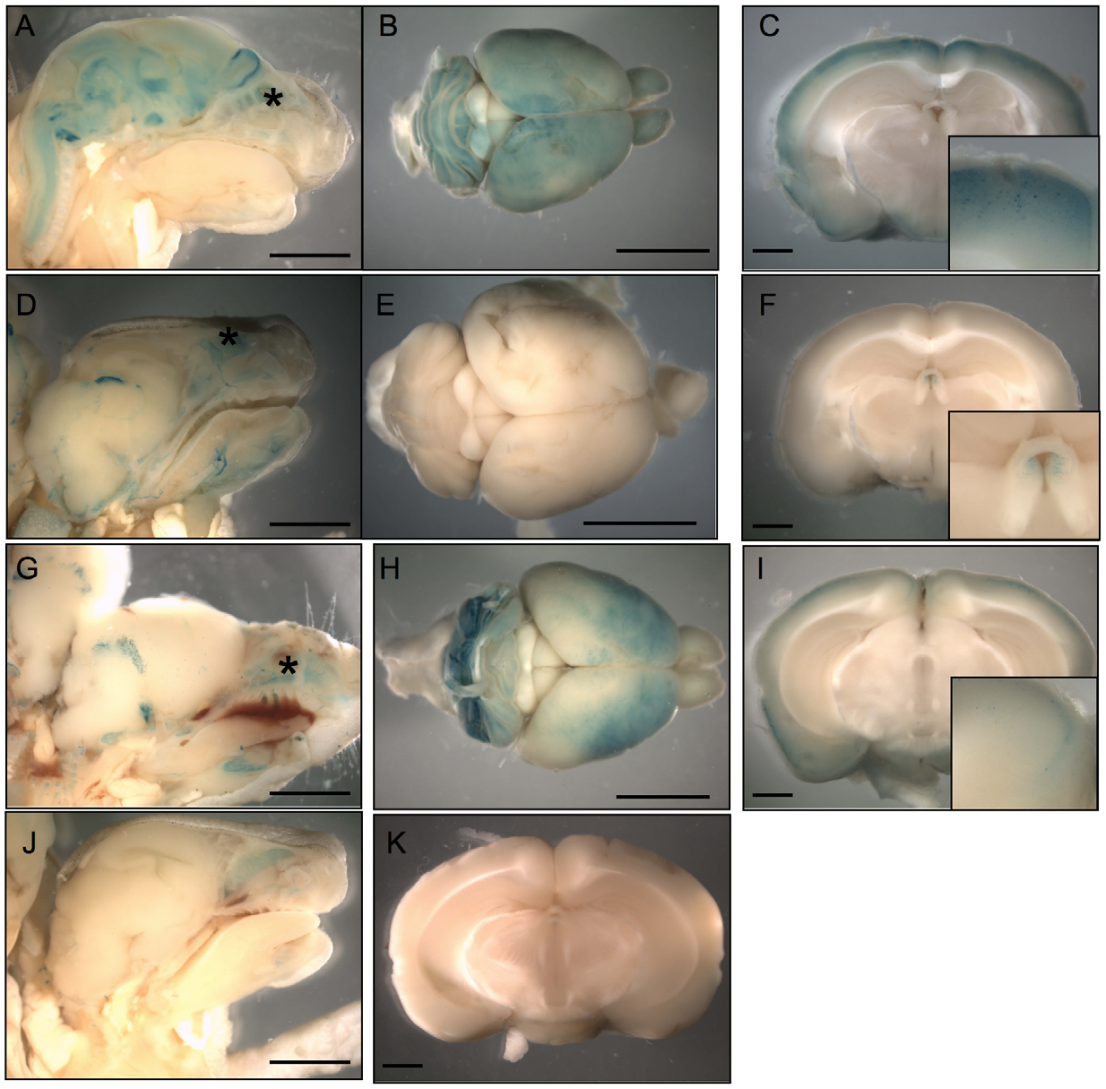
Brain-specific but variegated expression in newborn and adults from three different transgenic mouse lines. Newborn pups (A, D, G) or adult brains (B, C, E, F, H, I) from mouse transgenic lines 3 (A, B, C), 12 (D, E, F), and 21 (G, H, I) were stained for betagalactosidase activity, along with wild-type controls (J, K). Newborn pups (A, D, G, H) and adult brains (C, F, I, K) were cut sagitally or transversely, respectively, for X-Gal penetration and structure identification. Note the different patterns of expression in different mouse lines. Asterisks show a territory of endogenous, cytoplasmic betagalactosidase expression, the nasal pits, also seen in the wild-type controls (J). Scale bars: 5 mm (A, B, D, E, G, H, J) or 1 mm (C, F, I, K).

To further characterize the intronic promoter, we tested fiZ expression in newborn and adult mouse tissues (fig.5, table 1). Most tissues tested, including spinal cord, skeletal muscle, spleen, thymus, heart, lungs, liver, and oesophagus, were negative for LacZ expression. Staining, when observed, corresponded to tissues known to express high endogenous β-galactosidase activity, namely stomach, salivary glands, kidneys, and bones. Interestingly, 3/5 mouse lines showed expression in the newborn (fig. 5A, D, G) and adult brain (fig. 5B, C, E, F, H, I). Although they were not observed in non-transgenic animals fig. 5J, K), the structures stained were overlapping but different in each mouse line, suggesting that the intronic promoter is likely to contain some brain-specific regulatory elements that are insufficient to confer full specificity.

### Mapping of endogenous mRNA confirms that the intronic promoter is functional *in vivo*

**Figure 6:**
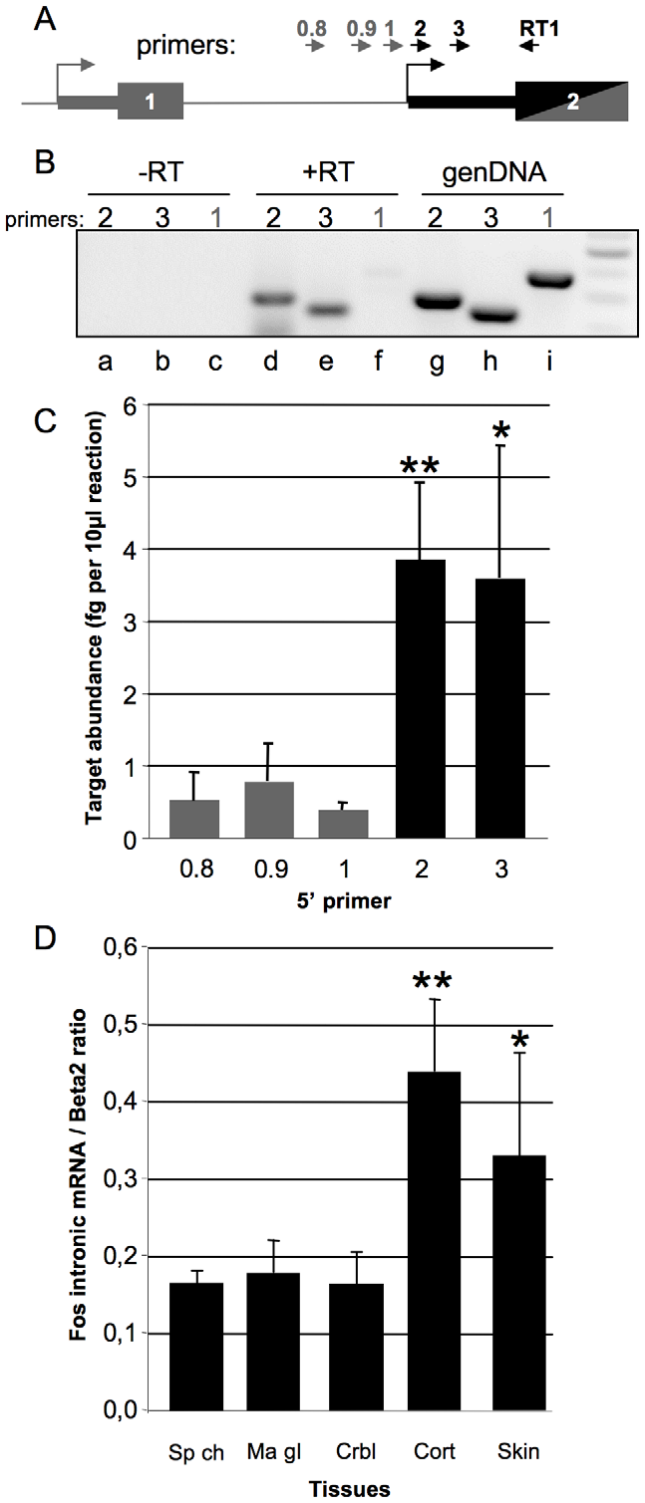
Validation of the expression profile and start site region of c-*fos* intronic mRNA by RT-QPCR. A. Schematic representation of the primers used in this RT-PCR study. Boxes represent exons, larger boxes are translated regions, arrows depict primers. The 3′ PCR primer was always RT1. 5′ primers in grey lie upstream the putative TSS, whereas primers in black are downstream. B. RT-PCR mapping of the c-*fos* intronic mRNA start site region. RNA extracted from E12.5 mouse embryo mammary buds was reverse transcribed with primer RT1, then cDNAs were amplified with primers used for detection of the intronic mRNA (primer 2 lane d, primer 3 lane e) and canonical pre-mRNA (primer 1, lane f). Minus RT control ensures that no genomic DNA contaminant was amplified with any primer (-RT: lanes a, b, c), while PCR on genomic DNA (genDNA: lanes g, h, i) shows that primer 1 is PCR-competent (lane i). The DNA ladder (last lane) shows fragments of 600, 500, 400, 300, and 200 bp. n = 4. C. Quantitative PCR-mediated mapping of the intronic mRNA start site on cDNA from adult mouse cortex. The five different 5′ primers described earlier were used along with RT1 for amplification. Signal was normalized according to standard curves elaborated for each primer pair, allowing a measure of the targeted RNA independently of primer efficiency. The scale shows the amount of RNA in fg per 10 µl of reaction. Error bars represent standard deviation, n = 4. D. RT-QPCR analysis of *fos* intronic mRNA expression in adult tissues. Quantitative PCR was ran with primers 2 and RT1 on cDNA prepared from dissected candidate adult mouse tissues (from left to right: spinal chord, mammary gland, cerebellum, cortex, skin). Normalization was done over the expression of beta-2 microgobulin. Error bars represent standard deviation, n = 4. Statistical analysis of variance (ANOVA) was performed relative to primer 0.8 (C) or to spinal chord level (D). *: p<0.05; **: p<0.01.

To confirm that LacZ expression in fiZ transgenic embryos really reflects an endogenous promoter activity, we performed RT-PCR on total RNA from developing mammary gland tissue dissected from wild-type E12,5 embryos (fig. 6B). We amplified fragments of the expected sizes (257 and 213 bp, respectively) with primers 2 and 3 (lanes d and e), located dowstream the putative start site. These fragments actually correspond to an endogenous RNA, since they were not obtained without reverse transcription (lanes a and b). To rule out any amplification of residual pre-mRNA from the canonical promoter, we used primer 1 (shown in red in fig. 1B), located upstream of the putative intronic start site. This primer yielded a very weak band (lane f), while it efficiently amplified genomic DNA (lane i). These data indicate that in the developing mammary bud, there is an endogenous mRNA that starts in c-*fos* first intron between positions +740 and +819 relative to the canonical start site. To confirm the result with primer 1, we used two additional primers (primers 0.8 and 0.9) located 126 and 30 bp upstream, respectively (fig. 6A). Using RT-qPCR on adult mouse cortex RNA, normalized by the relative efficiencies of the different primers on a cloned c-*fos* gene, primers 0.8, 0.9 and 1 similarly gave very weak signals, i.e. amplified a very low abundance species (fig. 6C). This confirms that they are targeting c-*fos* pre-mRNA rather than the intronic mRNA. The latter was readily detected by primers 2 and 3, thus placing the cryptic TSS between positions +740 and +819 relative to the canonic TSS.

To further analyze tissue-specific expression of the endogenous c-*fos* intronic RNA, we performed RT-QPCR on RNA extracted from various adult mouse tissues (fig.6D). Consistent with the transgenics data, expression was high in the adult cortex and low in the spinal chord. The adult mammary gland and cerebellum express low levels, while skin seemed to express intermediate levels. These data confirm the existence of a transcript starting in c-*fos* first intron *in vivo*, in tissues consistent with the transgenic analysis.

## Discussion

In this work, we describe a region of c-*fos* first intron that has been conserved throughout vertebrate evolution. This region contains TRE and CRE sites that are functional *in vitro*. In transiently transfected fibroblasts, it drives luciferase expression in the absence of any other promoter, which qualifies it as a genuine promoter. Importantly, this novel promoter is also active in transgenic developing embryos and adult brain, and has a very restricted expression pattern: only the mesenchymal part of the developing mammary bud and some CNS structures express the fiZ transgene. Finally, we confirm that an endogenous transcript resulting from intronic promoter activity exists in embryonic and adult tissues and starts in the intronic, conserved region.

Strikingly, c-*fos* first intron was previously pinpointed as one of the few untranslated regions that were highly conserved through evolution (our unpublished observation and ACUTS database: see [34]). While at that time the reason for its conservation was unclear, we now propose that it is because it performs a novel, tissue-specific promoter function for the gene.

One report seems to rule out the contribution of intragenic sequences in calcium- and growth factor-induced c-*fos* transcription [35]. Since the latter study was based on transiently transfected constructs, these discrepancies could be due either to the different cell types used or to a requirement for chromatin structure that is not reproduced on extra-chromosomal templates.

In contrast, several recent reports suggest that c-*fos* first intron improves reporter induction by calcium-mobilizing agents when appended to the upstream promoter ([36]–[39] and our unpublished observations). While this effect does not seem to be related to elongation control, it has not been established by which mechanisms this region affects overall trancription. Our results suggest that the enhancing effect measured may in fact be due to the sum of both canonical and intronic promoter activities.

The relatively high basal activity of the intronic promoter in transiently transfected fibroblasts (fig. 3B) contrasts with its highly restricted pattern in transgenic animals (figs. 4 and 5). This could indicate either the lack of chromatin environment in transient transfection, or the lack of some molecular determinants in the fiZ transgene for full widespread expression. Given the difficulties we experienced trying to detect the endogenous messenger in most tissues, we favour the first explanation. However, the temporal pattern of expression as seen in transgenic mice should be confirmed on the endogenous target at different stages of development (not only in adult cortex and e12.5 mammary glands), since it could be influenced by sequences near the insertion site.

Interestingly, in whole-mount embryos the continuous line of stained spinal cord cells extends from the hindbrain to the caudal region. Only one of the known motor columns has such an extended repartition, the others being restricted to limited segments along the rostro-caudal axis [40]. Thus it appears that the fiZ-expressing cells belong to the medial part of the medial motor column (MMCm), known to contain motor neurons innervating the axial muscles [41]. This pattern is reminiscent of that observed by Caubet [42], where a c-*fos* exon 4 probe, revealed a more widespread expression in spinal cord and brain of E14 embryos than we found. This discrepancy might be due to the fact that he was detecting expression of both promoters, and/or that our construct lacks sequences important for whole CNS expression. Consistent with the latter explanation, we find that the preferential CNS expression showed integration site-dependent effects. Interestingly, c-*fos*−/− mice were shown to have defects related to the CNS: apoptosis in the retina [43], altered spatial learning [44], and an impaired long-term response to kindling [45].

While our data add a novel level of complexity to c-*fos* transcription regulation, it is somewhat surprising that this feature was not discovered before. This can be explained both by the similar size of the predicted intronic mRNA and the canonic c-*fos* mRNA, and its low expression level, which forced us to resort to PCR-based methods for detection.

The predicted reading frame for this mRNA gives a protein starting at methionine 111 of canonical c-Fos. The first 110 aminoacids of c-Fos contain two Fos activation modules (FAMs), an inhibitor domain (ID1), and sequences necessary for cell transformation and negative cross-talk between AP-1 and the Glucocorticoid Receptors (GR) [46]. However, using antibodies to the N- and C-terminal regions of c-Fos, we were unable to detect c-FosΔN protein in adult tissue protein extracts. Nevertheless, it transactivates a 3xTRE-Luciferase reporter to the same extent as the full-length c-Fos protein (Coulon V. and Blanchard J. M., unpublished), suggesting that the functional differences between both isoforms might be more subtle. For example, the lack of interaction with a partner such as the GR would allow c-FosΔN to be insensitive to its negative control. Interestingly, the GR performs multiple functions in mammary gland development and cancer, some of them independent of DNA binding [47], including mammary gland differentiation in lactating female mice [48]. This alternate isoform of c-Fos would thus be insensitive to trans-regulation by corticoids. c-FosΔN also lacks a domain required for cell transformation; it could thus perform some of the functions of canonical c-Fos, such as transactivation, but would be unable to trigger cell transformation. Altogether, the tissue-specific expression of the intron-driven RNA and the primary structure of this novel Fos protein suggest a new function for the c-*fos* proto-oncogene.

## Materials and Methods

### DNA Sequences and Sequence Analysis

Alibaba, NNPP, BLAST and Clustal programs were used on their respective internet sites (http://wwwiti.cs.uni-magdeburg.de/~grabe/alibaba2/, http://www.fruitfly.org/seq_tools/promoter.html, http://www.ncbi.nlm.nih.gov/BLAST/and http://www.ebi.ac.uk/clustalw/). Sequences accession numbers for c-*fos* gene: *Homo sapiens* (VO1512), *Sus scrofa* (AJ132510), *Mus musculus* (V00727), *Xenopus laevis* (described in [32]), *Gallus gallus* (M18043), *Danio rerio* (NC_007131), *Fugu rubripes* (ENSTRUG00000010579).

### Reagents

DMEM tissue culture medium, penicillin, streptomycin, glutamine, and random primers DNA labeling system were obtained from Life Technologies, Inc. (Cergy Pontoise, France). Phorbol Myristate Acetate (PMA, used at 0.1 µg/ml), forskolin (used at 50 µM), A23187 (used at 5 µg/ml), X-Gal, potassium ferricyanide and ferrocyanide were purchased from Sigma-Aldrich (St. Quentin Fallavier, France). Radioactive nucleotides were from Amersham Pharmacia Biotech. All products for non-radioactive in situ hybridization came from Roche (Meylan, France). Cryosectionning materials were from Electron Microscopy Sciences (Euromedex, Mundolsheim, France) and CML (Nemours, France). Dual Luciferase Reporter Assay and Riboprobe system were purchased from Promega France.

### Plasmid Constructs

The fiL (*fos* intron Luciferase) plasmid was constructed by amplifying by PCR part of c-*fos* first intron with oligonucleotides 5′-actacgaggcgtcatcctcc-3′ and 5′-gcaagtggccctagtgtcgc-3′, and cleaving the PCR product with XhoI. The resulting fragment (+579 to +980 relative to the start site) was cloned in pGL2-basic vector (Promega). The SV40 promoter in pCH110 (Invitrogen) was deleted from SphI site to KpnI site, and replaced by a cassette encoding the SV40 NLS (oligonucleotides 5′-cctcgagcccgggaagctttctagaatg-gctccaaaaaagagaaaggtaccgg-3′ and 5′-ccggtacctttctcttcttttttggagccattctagaaagcttccc-gggctcgaggcatg-3′). This procedure yielded the pL-NLSLacZ plasmid. The fiZ plasmid (*fos* intron lacZ) was the result of cloning the XhoI-BglII fragment of c-*fos* gene (+579 to +1058) in the promoter-less, NLS-containing pLNLSLacZ vector. Expression vectors for AP-1 and CREB factors (maps available upon request) were constructed by cloning the respective cDNAs in the pcDNA3 vector (Invitrogen).

### Electro Mobility Shift Assays

EMSA were done according to [49]. Briefly, a Hela cell nuclear extract prepared as described [50] was incubated with a radioactive, double-stranded probe containing both the TRE and CRE sites from c-*fos* intron 1 (probe TRECRE, oligonucleotides: 5′-ggcgcctgcgtcagcgcagacgtcaggga-3′ and 5′-ggtccctgacgtctgcgctgacgcaggcg-3′) and labelled by klenow enzyme and 32P dCTP. Mutated oligonucleotide cassettes were used for competitions: mTRECRE (oligonucleotides: 5′-ggcgcctaagcttgcgcagacgtcaggga-3′ and 5′-ggtccctgacgtctgcgcaagcttaggcg-3′), TREmCRE (oligonucleotides: 5′-ggcgcctgcgtcagcgcaggatccaggga-3′ and 5′-ggtccctggatcctcgcgtgacgcaggcg-3′), mTREmCRE (oligonucleotides: 5′-ggcgcctaagcttgcgcaggatccaggga-3′ and 5′-ggtccctggatcctgcgcaagcttaggcg-3′).

Anti-ATFa monoclonal antibody (2F10) was described [51]. Antibodies to ATF-1, CREM-1 and to Fos and Jun families were purchased from Santa Cruz Biotech. Anti-CREB-1 was from New England Biolabs. Antibodies and non-radioactive competitors were incubated with the Hela cell extract for 15 min at room temperature before adding the probes for another 15 min.

### Transfection and cell culture

NIH3T3 (ATCC CRL-1658™), CCL39 (ATCC CCL-39™) and MEF cells were cultured at 37°C in a 5%CO2 incubator, in Dulbecco's modified Eagle's medium containing 10% fetal calf serum. MEFs were prepared from Black6 13.5 d.p.c. mouse embryos by trypsin digestion according to national regulations. Transfection was performed using the calcium phosphate method. 25 ng expression vector and 500 ng of reporter were usually used for each 35 mm well. Luciferase experiments were normalized to the activity of Renilla Luciferase, after co-transfection of the tk-RL plasmid (Promega) as described [52]. Measurements were done with a Lumat LB9501 Luminometer (Berthold).

### Transgenic Analysis

Animal care was performed according to national regulations and experiments approved by the regional ethics committee of Languedoc-Roussillon, France. Transgene DNA was separated from plasmid sequences by restriction digestion and gel electrophoresis, purified on a sucrose gradient and injected (2 ng/ml) into the pronuclei of fertilized embryos of (C57 Bl/6 CBA) F1 mice (from Iffa-Credo, France). After incubation overnight, two-cell embryos were transferred to the oviducts of pseudopregnant foster mothers. Animals were genotyped by PCR using tail DNA and 2 primers hybridizing respectively to c-*fos* first intron and LacZ cDNA (5′-gcgagttcattctggagact-3′ and 5′-gtaaaacgacgggatcgatc-3′). PCR genotyping was confirmed by southern blot analysis using a ^32^P-labeled LacZ probe (random priming method, see [22]). Male founders were mated to wild-type (C57 Bl/6 CBA) F1 females that were then sacrificed by cervical disruption at different times post-conception. Embryos were dissected out from uterus and yolk sac, fixed 20 min in 4% Paraformaldehyde, rinsed in PBS (containing NP-40 0.01% and deoxycholate 0.01% for embryos older than E12 and adult tissues), and incubated overnight at 30°C, pH 7.4 in PBS containing 0.4 mg/ml X-Gal, potassium ferricyanide and ferrocyanide (4 mM each), and 4 mM MgCL_2_ (as described in [53]). Embryos were either photographed with a stereomicroscope and digital color camera, or sectionned in a cryotome, and the sections counterstained with eosin, before photographing.

### RT-PCR analysis

Total RNA was prepared from dissected mouse embryos or adult tissues with TRIZOL reagent (Invitrogen) according to the manufacturer's directions, except that frozen tissues were homogenized with a FastPrep®-24 instrument (MP Biomedicals) and zirconium beads before phase separation. RT was performed on 1 µg total RNA with 200 U superscript II enzyme (Invitrogen) and a c-*fos* exon2 primer (RT1, 5′-actagagacggacagatctg-3′) or random hexamers before DNase I (Roche) treatment and column purification (nucleospin, Macherey-Nagel). For PCR, we used 1 tenth of the RT reaction, and the oligonucleotides used were RT1 and one of the following: primer 0.8 (5′-gctaactagagtttgggagg-3′), primer 0.9 (5′-gggtgtgtaaggcagtttca-3′), primer 1 (5′-gcgagttcattctggagact-3′, red in fig. 1B), primer 2 (5′-ataacgggaacgcagcagta-3′, in green in fig.1B) or primer 3 (5′-tgcggaattcaagggaggat-3′). Quantitative PCR was ran on a Light Cycler 480 apparatus (Roche) with Taq platinum (Invitrogen) and a SYBR Green mix containing 3 mM MgCl2 and dNTPs 30 µM each. qPCR conditions were 45 cycles as follows: 95°C for 4 s, 62°C for 10 s, and 72°C for 30 s. Normalization was done according to beta-2 microglobulin housekeeping gene (selected for its constant expression in the tissues tested), or according to a standard dilution curve on a c-*fos* mouse gene-containing plasmid when comparing targets amplified with different primers.
